# Heated tobacco product use and its relationship to quitting combustible cigarettes in Korean adults

**DOI:** 10.1371/journal.pone.0251243

**Published:** 2021-05-07

**Authors:** Jinyoung Kim, Sungkyu Lee, Heejin Kimm, Juna-Ah Lee, Cheol-min Lee, Hong-Jun Cho

**Affiliations:** 1 Southern Gyeonggi Regional Smoking Cessation Centre, Hallym University, Anyang, Republic of Korea; 2 Korea Tobacco Control Research and Education Centre, Seoul, Republic of Korea; 3 Department of Epidemiology and Health Promotion, Institute for Health Promotion, Graduate School of Public Health, Yonsei University, Seoul, Republic of Korea; 4 Department of Family Medicine, Asan Medical Centre, University of Ulsan College of Medicine, Seoul, Republic of Korea; 5 Department of Family Medicine, Healthcare System Gangnam Center, Seoul National University Hospital, Seoul, Republic of Korea; University of Calfornia San Francisco, UNITED STATES

## Abstract

**Objective:**

We assessed the prevalence of, and factors associated with, heated tobacco product (HTP) use and analysed the association between HTP use and quitting combustible cigarettes (CCs) in Korean adults.

**Methods:**

We conducted an online survey with 7,000 adults (males, 2,300; females, 4,700; ages 20–69) out of 70,000 age-, sex- and provincial-distribution-matched individuals based on 2018 national population statistics. Females were oversampled because the prevalence of tobacco product use is very low among women in Korea. Chi-square tests were used for bivariate analyses, and odds ratios were assessed after adjusting for sociodemographic variables.

**Results:**

The prevalence of current CC, electronic cigarette (EC), and HTP use was 24.8% (males, 40.4%; females, 9.3%), 6.8% (males, 10.1%; females, 3.4%), and 10.2% (males, 16.2%; females, 4.3%), respectively. Among the 574 current HTP users, 77 (13.4%) were HTP-only users and >80% were either dual users of HTP and CC/EC, or triple users of HTP, EC, and CC. Among the current CC users, the odds of having attempted to quit CCs in the past year were greater among EC-only users (aOR 2.92; 95% CI 1.81–4.69) and dual users of HTPs and ECs (aOR 8.42; 95% CI 4.85–14.62) than among non-HTP and non-EC users. Among 2,121 ever CC smokers, the likelihood of being a former CC smoker was 0.19 (95% CI 0.15–0.24) for HTP users, 0.29 (95% CI 0.20–0.42) for EC users, and 0.03 (95% CI 0.01–0.06) for users of both HTPs and ECs compared with non-HTP and non-EC users.

**Conclusion:**

EC-only use and dual use of HTPs and ECs were associated with increased attempts to quit CCs; however, HTP and EC use was associated with lower odds of CC smoking abstinence.

## Introduction

Heated tobacco products (HTPs) are tobacco products that produce aerosols containing nicotine and other chemicals, which are inhaled by users [1]. According to a systematic review, HTPs have at least 62% lower levels of harmful and potentially harmful toxicants than combustible cigarettes (CCs) [2]. On July 7, 2020, the US Food and Drug Administration (US FDA) authorised marketing of IQOS, an HTP produced by Philip Morris International. The US FDA stated that IQOS significantly reduces the production of harmful and potentially harmful chemicals (HPHC) and reduces the body’s exposure to HPHC when switching completely from conventional cigarettes to IQOS [3]. However, the US FDA did not accept the claims of reduced health risks made by tobacco companies because there are no data on the long-term effects of HTPs on health. Philip Morris International introduced IQOS to the Japanese market in 2014 and the Korean market in 2017. Since then, the market share of HTPs in Korea has grown rapidly [4]. Additional HTP products (Glo, manufactured by the British American Tobacco Company, and Lil, manufactured by KT&G) are now available in Korea. The market share by sales of HTPs in Korea reportedly increased from 6.1% in December 2017 to 11.8% in April 2019 [5]. In Japan, IQOS had close to 15% of the national tobacco market share in 2018 [6]. However, the market growth has been faster in Korea than in Japan [7, 8]. Except for tobacco tax levels on HTPs, which are 89% of those on CCs, HTPs are currently regulated at the same level as CCs; use of HTPs in public places and their sale to minors is banned, advertisement is regulated, and pictorial warning labels are applied to HTP sticks [9].

Few studies have evaluated the current and ever use of HTPs. A study in Korea 3 months after IQOS entered the market found that the prevalence of current HTP use among Korean young adults was 3.5% [4]. In Japan, the prevalence of HTP use was 0.6% in 2015 and 3.6% in 2017. In Italy, the percentage of those ever having used HTPs was 3.1% among current CC smokers and 7.7% among current EC users [8].

One of the reasons for using HTPs among CC smokers was to quit CCs [10]; however, whether or nor HTPs could help CC smokers quit smoking remains unknown. One cross-sectional study involving Korean adolescents who were ever users of CCs reported that HTP use had a low probability of making them quit CCs [11].

Some studies have evaluated the factors associated with HTP use. In Japan, HTP use was more prevalent among men, among individuals aged 20–29 years, among high-income individuals, and among individuals with high educational levels [7]. In Hong Kong, the ever use of HTPs was more prevalent among individuals aged 30–39 years, among high-income individuals, and among individuals with high educational levels [12]. A qualitative study in Japan and Switzerland found that adult IQOS users considered IQOS to be clean, chic, and pure. Moreover, they found IQOS suitable for use indoors or in non-smoking areas because it did not produce ash and produced considerably less of an odour than CCs [13].

The prevalence of CC use in Korean adult males was almost 80% in the 1980s; however, it had decreased to 38.1% by 2017 [14]. The prevalence of CC use in Korean adult females was considerably lower at 5%–7% during the same period [14]. ECs were introduced to the Korean market in 2008, and in 2017, the prevalence of EC use among Korean adults was 4.4% among men and 0.9% among women [14].

In this study, we investigated the prevalence of, and factors associated with, HTP use among adults in Korea. We compared attempts to quit by HTP/EC users with HTP/EC non-users among current CC smokers and the odds of abstinence of CC use for HTP/EC users with HTP/EC non-users among ever users of CCs.

## Materials and methods

### Study sample and procedures

We conducted an online survey using a sample from a group of individuals (termed a panel) managed by the commercial database company EMBRAIN (http://www.embrain.com/eng/) comprising 1,300,000 members as of December 2018. First, we randomly selected 70,000 members aged 20–69 years from the panel members via e-mail. They were selected to be age- and provincial-distribution-matched with 2018 national population statistics. Of the 70,000 members, 10,489 replied, and 7,783 answered the questionnaires (response rate, 11%). Of these 7,783 members, 7,000 members (2,300 mean and 4,700 women) were included in the analysis after excluding 2,238 who exceeded the quota, 21 who were outside the age range of the study subjects, and 400 who decided not to participate in the survey. We sampled twice as many women as men because very few women use tobacco products in Korea.

Data was collected between November 3rd and 9th, 2018. The questionnaire we used was developed as a part of this study, and the copyright for it belongs to the Ministry of Health in Korea, who funded this study. All participants received compensation equivalent to 5,000 Korean Won (KRW; approximately 5 USD).

This study was approved by the Institutional Review Board of the Asan Medical Centre (S2018-1662-0001). Informed consent was obtained from all individual study participants. The participants willing to participate in this survey were asked to read and sign the consent form before beginning the online survey.

### Measures

CC smoking was assessed by asking the following question: ‘Do you currently smoke cigarettes every day, some days, or not at all?’ Those who responded ‘every day’ or ‘some days’ with a lifetime use of ≥100 cigarettes were classified as current CC smokers. Those who responded ‘not at all’ with a lifetime use of ≥100 cigarettes were classified as former CC smokers. Those who reported smoking fewer than 100 cigarettes in their lifetime were classified as never having been CC smokers.

EC use was assessed by asking the following questions: ‘Have you ever used e-cigarettes in your life?’ (yes/no), and ‘Have you used e-cigarettes in the past 30 days?’ (yes/no). Those who had used ECs in their lifetime and in the past 30 days were classified as current EC users. Those who had previously used ECs but not in the past 30 days were classified as former EC users.

In addition, HTP use was assessed by asking the following question: ‘Have you ever used a heated tobacco product in your life?’ (yes/no). Those who answered ‘yes’ to this question were classified as ever HTP users. These individuals were asked if they currently used HTPs ‘every day’, ‘some days’, or ‘not at all’. Those who reported using HTPs ‘every day’ or ‘some days’ were classified as current HTP users, whereas those who responded ‘not at all’ were classified as former HTP users. To minimise the confusion in distinguishing between HTPs and ECs, HTP images and names (including IQOS, Glo, and Lil) were provided.

People who attempted to quit CC smoking were current CC smokers who answered ‘yes’ to the question ‘During the past 12 months, have you made attempts to quit combustible cigarette smoking?’

Demographic characteristics including sex, age, educational level, marital status, and monthly income were collected. Monthly incomes were categorised as follows: <3,000,000 KRW (1 USD = 1000 KRW), 3,000,000–4,999,999 KRW, 5,000,000–6,999,999 KRW, or ≥7,000,000 KRW. Education was categorised as high school level or lower or university degree or higher. Marital status was categorised as married or living with a partner, separated/widowed/divorced, or never married.

### Data analysis

All analyses were weighted considering the oversampling of females relative to males. Descriptive statistics are presented as unweighted numbers and weighted percentages. A logistic regression analysis was conducted to examine factors related to CC smokers’ quit attempts. The analysis provided a comparison between HTP or EC use among current CC smokers and HTP or EC use among those who have ever been CC smokers. The results are presented as odds ratios (OR) and 95% confidence intervals (CI), and adjusted OR (aOR) estimates were obtained for the aforementioned covariates. All statistical analyses were performed using SAS 9.4 (SAS Institute Inc., Cary, NC, USA) [15].

## Results

Among the 7,000 study participants, 24.8% were current CC users (males, 40.4%; females, 9.3%), 10.2% were current HTP users (males, 16.2%; females, 4.3%) and 6.8% were current EC users (males, 10.1%; females, 3.4%) (Table 1).

**Table 1:** General characteristics of study participants. (N = 7,000).

	Total		Male		Female	
	N	%	N	% ^a^	N	% ^a^
**Study population**	**7,000**	**100**	**2,300**	**50**	**4,700**	**50**
Location						
Provinces	3,781	54.3	1,268	55.1	2,513	53.5
Metropolitan cities	3,219	45.7	1,032	44.9	2,187	46.5
Age (years)						
20–34	2,127	30.3	690	30.0	1,437	30.6
35–49	2,445	35.3	833	36.2	1,612	34.3
50–69	2,428	34.5	777	33.8	1,651	35.1
Education						
≤High school	1,545	21.1	416	18.1	1,129	24.0
≥University	5,455	78.9	1,884	81.9	3,571	76.0
Marital status						
Never married	2,390	34.6	823	35.8	1,567	33.3
Married/living with a partner	4,309	61.4	1,399	60.8	2,910	61.9
Separated/widowed/divorced	301	4.1	78	3.4	223	4.7
Income (KRW)^b^						
<3,000,000	1,774	25.7	618	26.9	1,156	24.6
3,000,000–4,999,999	2,431	34.9	817	35.5	1,614	34.3
5,000,000–6,999,999	1,554	21.8	476	20.7	1,078	22.9
≥7,000,000	1,241	17.5	389	16.9	852	18.1
Combustible cigarette use						
Never	4,879	61.2	832	36.2	4,047	86.1
Former	757	14.0	539	23.4	218	4.6
Current	1,364	24.8	929	40.4	435	9.3
E-cigarette use						
Never	6,031	83.0	1,700	73.9	4,331	92.1
Former	575	10.2	368	16.0	207	4.4
Current	394	6.8	232	10.1	162	3.4
Heated tobacco product use						
Never	6,056	83.2	1,692	73.6	4,364	92.9
Former	370	6.5	235	10.2	135	2.9
Current	574	10.2	373	16.2	201	4.3

^a^ Values are presented as unweighted numbers and weighted percentages.

^b^ KRW, Korean Won (1,000 KRW = 1 USD).

Among the 1,530 current tobacco product users, current CC users were 789 (52.3%), followed by current dual users of CCs and HTPs (270, 18.4%), current triple users of CCs, ECs, and HTPs (194, 12.4%), and current dual users of CCs and ECs (111, 7.2%). Current HTP-only users and current dual users of ECs and HTPs were 77 (4.7%) and 33 (1.8%), respectively (Table 2). HTP users that also used other tobacco products (18.4% for dual users of CCs and HTPs, 1.8% for dual users of ECs and HTPs, and 12.4% for triple users of CCs, ECs, and HTPs) were much more prevalent than HTP-only users (4.7%) (Table 2).

**Table 2:** Patterns of tobacco product use among current tobacco product users (N = 1,530).

	Male (N = 1,031)	Female (N = 517)	Total (N = 1,530)
	N (%)	N (%)	N (%)
Current CC use only	541 (53.4)	248 (48.0)	789 (52.3)
Current HTP use only	44 (4.3)	33 (6.4)	77 (4.7)
Current EC use only	27 (2.7)	29(5.6)	56 (3.2)
Current use of both CCs and HTPs	196 (19.3)	74 (14.3)	270 (18.4)
Current use of both ECs and HTPs	13 (1.3)	20 (3.9)	33 (1.8)
Current use of both CCs and ECs	72 (7.1)	39 (7.5)	111 (7.2)
Current triple use of CCs, ECs, and HTPs	120 (11.8)	74 (14.3)	194 (12.4)

CC, combustible cigarette; EC, electronic cigarette; HTP, heated tobacco product.

Values are presented as unweighted numbers (weighted percentages).

Current users of CCs or ECs had greater odds of using HTPs [aOR (95% CI); 17.20 (13.79–21.47) and 27.84 (18.46–41.99), respectively] than non-CC and non-EC users (Table 3). The odds of using HTPs were much greater for those who used both ECs and CCs [aOR (95% CI); 87.22 (66.81–113.86)] than for individuals who used neither ECs nor CCs. The odds for EC users were greater than those for CC users [(aOR (95% CI); 1.45 (0.98–2.15)] when compared pairwise (data not shown). The odds were greater for males than females [aOR (95% CI); 1.52 (1.25–1.86)] and for individuals with an educational level of university or higher than for individuals with an educational level of high school or lower [aOR (95% CI); 1.77 (1.40–2.23)]. The odds of using HTP were lower for individuals aged 50–69 years than for individuals aged 20–34 years [aOR (95% CI); 0.49 (0.38–0.64)].

**Table 3:** Factors related to current HTP use. (N = 7,000).

Factor	Adjusted Odds Ratio	95% Confidence Interval
Sex		
Female	1.00	
Male	1.52	1.25–1.86
Age (years)		
20–34	1.00	
35–49	1.03	0.82–1.30
50–69	0.49	0.38–0.64
Education		
≤High school	1.00	
≥University	1.77	1.40–2.23
Marital status		
Never married	1.00	
Married/living with a partner	1.30	1.04–1.62
Separated/widowed/divorced	0.93	0.59–1.49
Income (KRW) ^a^		
<3,000,000	1.00	
3,000,000–4,999,999	1.09	0.87–1.37
5,000,000–6,999,999	1.52	1.18–1.95
≥7,000,000	1.42	1.09–1.84
Tobacco use status		
CCs (−) and ECs (−)	1.00	
CCs (+) and ECs (−)	17.20	13.79–21.47
CCs (−) and ECs (+)	27.84	18.46–41.99
CCs (+) and ECs (+)	87.22	66.81–113.86

Adjusted for sex, age, education, marital status, income, and tobacco use status.

aOR, adjusted odds ratio; CI, confidence interval; CCs, combustible cigarettes; ECs, electronic cigarettes; HTPs, heated tobacco products.

Values are presented as unweighted numbers (weighted percentages).

^a^ KRW, Korean Won (1,000 KRW = 1 USD).

(−), non-use; (+), use.

Among the 1,128 current CC smokers, the odds of having attempted to quit CC smoking in the past year were greater for dual CC and EC users [aOR (95% CI); 2.92 (1.81–4.69)] and triple users of CCs, HTPs, and ECs [aOR (95% CI); 8.42 (4.85–14.62)] than for individuals who only used CCs (Table 4).

**Table 4:** Factors related to CC smoking quit attempts in the past year among current CC smokers. (N = 1,128).

	Attempts to quit CCs at least once vs. no CC quit attempts	
	N(%) = 851(74.3%) N(%) = 277(25.7%)	
	Adjusted Odds Ratio	95% Confidence Interval
Sex		
Female	1	
Male	0.61	0.45–0.83
Age (years)		
20–34	1	
35–49	0.51	0.37–0.70
50–69	0.48	0.34–0.69
Education		
≤High school	1	
≥University	1.12	0.85–1.48
Marital status		
Never married	1	
Married/living with a partner	0.85	0.64–1.13
Separated/widowed/divorced	0.62	0.37–1.02
Income (KRW)^a^		
<3,000,000	1	
3,000,000–4,999,999	1.39	1.05–1.83
5,000,000–6,999,999	1.56	1.12–2.16
≥7,000,000	1.57	1.10–2.23
Tobacco use status		
HTPs (−) and ECs (−)	1	
HTPs (+) and ECs (−)	1.07	0.83–1.38
HTPs (−) and ECs (+)	2.92	1.81–4.69
HTPs (+) and ECs (+)	8.42	4.85–14.62
Numbers of cigarettes per day		
≤10	1	
11–19	0.53	0.41–0.68
≥20	0.64	0.49–0.85

Adjusted for sex, age, education, marital status, income, tobacco use status, and number of cigarettes per day.

aOR, adjusted odds ratio; CI, confidence interval; CCs, combustible cigarettes; ECs, electronic cigarettes; HTPs, heated tobacco products.

^a^ KRW, Korean Won (1,000 KRW = 1 USD).

(−), non-use; (+), use.

Among the 2,121 ever CC smokers, the likelihood of being a former CC smoker was 0.19 (95% CI 0.15–0.24) for HTP users, 0.29 (95% CI 0.20–0.42) for EC users and 0.03 (95% CI 0.01–0.06) for users of both HTPs and ECs compared with non-HTP and non-EC users (Table 5).

**Table 5:** Factors related to being a former CC smoker among ever CC users. (N = 2,121).

	Former CC^a^ users vs. current CC smokers among ever CC users	
	N(%) = 757(36.1%) N(%) = 1364(63.9%)	
Factor	Adjusted Odds Ratio	95% Confidence Interval
Sex		
Female	1.00	
Male	0.37	0.28–0.49
Age (years)		
20–34	1.00	
35–49	1.22	0.96–1.55
50–69	1.62	1.26–2.09
Education		
≤High school	1.00	
≥University	1.35	1.11–1.65
Marital status		
Never married	1.00	
Married/living with a partner	1.61	1.30–1.99
Separated/widowed/divorced	0.96	0.65–1.40
Income (KRW)^a^		
<3,000,000	1.00	
3,000,000–4,999,999	0.98	0.80–1.20
4,999,999–6,999,999	1.05	0.83–1.32
≥7,000,000	1.32	1.04–1.69
Tobacco use status		
HTPs (–) and ECs (–)	1.00	
HTPs (+) and ECs (–)	0.19	0.15–0.24
HTPs (–) and ECs (+)	0.29	0.20–0.42
HTPs (+) and ECs (+)	0.03	0.01–0.06

Adjusted for sex, age, education, marital status, income, and tobacco use status.

aOR, adjusted odds ratio; CI, confidence interval; CCs, combustible cigarettes; ECs, electronic cigarettes; HTPs, heated tobacco products.

^a^ KRW, Korean Won (1,000 KRW = 1 USD).

(−), non-use; (+), use.

## Discussion

Dual users of CCs and ECs and triple users of CCs, HTPs, and ECs were more likely to have attempted to quit CC smoking in the past year when compared with CC-only users. However, among those ever having been CC smokers, the odds of being former smokers were much lower among dual users of CCs and HTPs or ECs and triple users of HTPs, ECs, and CCs than among non-HTP and non-EC users. Among adult CC smokers, those who used ECs were more likely to have attempted to quit CC smoking [16, 17], which was also the case with adolescent CC smokers [18] because quitting CC smoking is an important reason for using ECs, particularly among adults [19]. However, there is controversy concerning the effect of EC use on CC smoking cessation. Several clinical trials and observational studies have shown that EC use increased CC smoking cessation [20–22]; however, a systematic review showed that EC use reduced the odds of quitting CC smoking [23]. There is a paucity of evidence concerning the effects of HTP use or dual use of HTPs and ECs on attempts to quit CCs and CC smoking cessation. Among Japanese adults, current CC smokers with the intention to quit were more likely to use IQOS than those who did not intend to quit CC smoking [7]. When comparing current adolescent Korean CC smokers who had ever used HTPs with those who had never used HTPs, there were fewer former CC smokers among those who had ever used HTPs [11]. These findings imply that HTP use, particularly in conjunction with EC use, may reduce CC smoking cessation. Because we do not have information about the dates of the last cigarettes for former CC smokers, we are unsure whether HTP use contributes to reduced CC smoking cessation; moreover, another possible explanation is that those who do not intend to quit CCs may be prone to using HTPs. We need to perform longitudinal studies to determine which explanation is more plausible.

In our study, the prevalence of current HTP users was 10.2% (males, 16.2%; females, 4.3%). Among current HTP users, >80% were either dual HTP and CC users or dual HTP and EC users (52.7%) or triple users of HTPs, CCs, and ECs (33.8%). These findings are similar to those of a previous study in Japan that showed 72% of current HTP/EC users used CCs concurrently [7]. Changes in tobacco product use from single to multiple product have several public health implications. First, this type of change goes against the tobacco industry’s recommendation of simply completely switching from CCs to HTPs [24, 25] and the assumption that reduced HPHS exposure will result from a complete switch [26]. Second, claims of reduced health risk related to HTP use cannot be sustained because cardiovascular risks associated with CC smoking are not linear but exponential, which means that a reduction in the amount of CC smoke does not proportionally reduce cardiovascular risk [27], and lung cancer risk is more dependent on CC smoking duration rather than the amount of CCs smoked [28]. Third, nicotine exposure and dependence symptoms can be stronger in individuals who use multiple tobacco products than in individuals who use single tobacco products [29, 30]. Strong nicotine dependence is an important barrier to successful CC smoking cessation [31]; therefore, the use of multiple tobacco products may hinder CC smoking cessation. Fourth, multiple tobacco product users can easily overcome the inconvenience of public-place smoking bans and do not experience social pressure to quit CC smoking because HTPs produce no ash, odour, or visible smoke and are more accepted socially [13]. Fifth, CC smokers of higher socioeconomic status are more likely to use HTPs than CC smokers of lower socioeconomic status, who form the majority of CC smokers. This means there is less chance of reducing population-level harm caused by CC smoking by positioning HTPs as a CC alternative. In addition, the finding that current EC users had higher odds of HTP use than current CC users also suggests that HTPs may not reduce the population-level harm caused by CCs. Finally, users of multiple tobacco products perceive the harmfulness of tobacco products less than users of single tobacco products [32].

The existence of multiple tobacco products, including HTPs, is a new challenge to tobacco control policy, which mainly focuses on CCs. Like CCs, HTPs and ECs are regulated as tobacco products in Korea. However, the tax levied on HTPs is 89% of that levied on CCs, and HTP devices are not regulated as tobacco products, which may contribute to their popularity in Korea. Tobacco control policy must adapt to this changing situation and the development of multiple tobacco products.

In this study, factors associated with greater odds of HTP use were male sex, young age, higher educational level and income, being married, and being an EC and/or CC user. HTPs have attracted CC smokers because it was advertised as being less harmful and producing less odour or smoke. Moreover, it has been popular among young Korean men of higher socioeconomic status because this group was more likely able to adapt this novel product earlier [33]. Considering the lower prevalence of CC use among individuals of lower socioeconomic status in Korea [34], the popularity of HTPs in higher socioeconomic status individuals may hamper efforts to reduce the population-level harm associated with tobacco use. Notably, CC or EC use is the most important factor determining HTP use in Japan, the UK, the USA, Hong Kong, and Italy [7, 8, 12, 35, 36], and HTP use is more prevalent among younger individuals in Japan, the UK, Hong Kong, and Italy. There is no consistent socioeconomic difference in HTP use in some countries, including Japan, the UK, Hong Kong, and Italy.

### Limitations

This study had some limitations. First, the sample of the present study was not a national representation, and the response rate was low (11%); therefore, the prevalence of HTP use should be interpreted with caution. However, the relationship found between tobacco products and sociodemographic factors is valid. Further studies with nationally representative samples must be conducted. Second, there may have been some confusion between the terms HTPs and ECs among survey participants because HTPs are novel tobacco products, and the names of HTPs (“cigarette-type e-cigarette”) and ECs (“liquid-type e-cigarette”) are similar in Korean. To reduce this confusion, we asked questions concerning ECs first and provided pictures and names of HTP products examples. Third, we did not include the dates of last cigarettes for former CC smokers. Therefore, the rate of quit attempts in the last year may have been underestimated. Moreover, it is not clear whether being a forms CC smoker is after using HTPs. Finally, because this was a cross-sectional study, causality cannot be concluded. A longitudinal study is needed to infer causality.

## Conclusions

Approximately 10% of the Korean adults sampled were current HTP users. Most HTP users also used CCs and/or ECs. Among individuals who had smoked CCs in their lifetime, the use of ECs and the dual use of HTPs and ECs was related to an increased likelihood of CC quit attempts; however, the use of HTPs or ECs and the dual use of HTPs and ECs reduced their odds of abstaining from CC smoking. Korean tobacco control policies should now aim to address this new challenge.

## Supporting information

S1 Table Patterns of tobacco product use among current tobacco product users (N = 1530). (DOCX)
