# Correction: Heated tobacco product use and its relationship to quitting combustible cigarettes in Korean adults

**DOI:** 10.1371/journal.pone.0296234

**Published:** 2023-12-19

**Authors:** Jinyoung Kim, Sungkyu Lee, Heejin Kimm, Juna-Ah Lee, Cheol-min Lee, Hong-Jun Cho

The fourth author’s name is spelled incorrectly. The correct name is: Jung-Ah Lee. The correct citation is: Kim J, Lee S, Kimm H, Lee J-A, Lee C-m, Cho H-J (2021) Heated tobacco product use and its relationship to quitting combustible cigarettes in Korean adults. PLoS ONE 16(5): e0251243. https://doi.org/10.1371/journal.pone.0251243.
