# Supplementary material for: Heated tobacco product use and its relationship to quitting combustible cigarettes in Korean adults
Source: PLoS One. 2021 May 7;16(5):e0251243. doi: 10.1371/journal.pone.0251243 (PMC8104442; doi:10.1371/journal.pone.0251243)
Supplement: S1 Table — (DOCX) [file pone.0251243.s001.docx]

**S1 Table. Patterns of tobacco product use among current tobacco product users (N = 1530)**

|  | **Male**  **(N = 1031)** | **Female**  **(N = 517)** | **Total**  **(N = 1530)** |
| --- | --- | --- | --- |
|  | N (%) | N (%) | N (%) |
| Current CCs use only | 541 (53.4) | 248 (48.0) | 789 (52.3) |
| Current HTP use only | 44 (4.3) | 33 (6.4) | 77 (4.7) |
| Current ECs use only | 27 (2.7) | 29(5.6) | 56 (3.2) |
| Dual current use of CCs and HTPs | 196 (19.3) | 74 (14.3) | 270 (18.4) |
| Dual current use of ECs and HTPs | 13 (1.3) | 20 (3.9) | 33 (1.8) |
| Dual current use of CCs and ECs | 72 (7.1) | 39 (7.5) | 111 (7.2) |
| Triple current use of CCs, ECs and HTPs | 120 (11.8) | 74 (14.3) | 194 (12.4) |

CC, combustible cigarette; EC, electronic cigarette; HTP, heated tobacco product

Values are presented as unweighted numbers (weighted percentages).
